# Prognostic significance of the controlling nutritional status (CONUT) score in patients undergoing gastrectomy for gastric cancer: a systematic review and meta-analysis

**DOI:** 10.1186/s12893-019-0593-6

**Published:** 2019-09-05

**Authors:** Kosei Takagi, Piotr Domagala, Wojciech G. Polak, Stefan Buettner, Bas P. L. Wijnhoven, Jan N. M. Ijzermans

**Affiliations:** 1 Department of Surgery, Erasmus MC, University Medical Center Rotterdam, Dr. Molewaterplein 40, 3015 GD Rotterdam, The Netherlands; 2 Department of Gastroenterological Surgery, Okayama University Graduate School of Medicine, Dentistry, and Pharmaceutical Sciences, Okayama, Japan; 3 Department of General and Transplantation Surgery, The Medical University of Warsaw, Warsaw, Poland

**Keywords:** Gastric cancer, Controlling nutritional status (CONUT) score, Gastrectomy, Outcome, Meta-analysis

## Abstract

**Background:**

In recent years, the clinical evidence of the controlling nutritional status (CONUT) score has increased in patients with gastrointestinal cancers. The purpose of this systematic review and meta-analysis was to investigate the association between the preoperative CONUT score and outcomes in patients undergoing gastrectomy for gastric cancer (GC).

**Methods:**

A systematic literature search for studies reporting the prognostic impact of the CONUT score in patients with GC was conducted. Meta-analyses of survival, postoperative outcomes, and postoperative clinico-pathological parameters were conducted.

**Results:**

Five studies with 2482 patients were found to be eligible and subsequently reviewed and analyzed. The CONUT score was significantly associated with overall survival (HR 1.85, 95%CI 1.38–2.48, *P* <  0.001), cancer-specific survival (HR 2.56, 95%CI 1.24–5.28, *P* = 0.01) and recurrence/relapse-free survival (HR 1.43, 95%CI 1.12–1.82, *P* = 0.004). Moreover, the CONUT score was associated with the incidence of postoperative complications (OR 1.39, *P* = 0.003) and mortality (OR 6.97, *P* = 0.04), and clinico-pathological parameters (T factor [OR 1.75, *P* <  0.001], N factor [OR 1.51, *P* <  0.001], TNM stage [OR 1.73, *P* <  0.001], and microvascular invasion [OR 1.50, *P* = 0.006]), but not with tumor differentiation (OR 0.85, *P* = 0.13).

**Conclusions:**

The preoperative CONUT score is an independent prognostic indicator of survival and postoperative complications, and is associated with clinico-pathological parameters in patients with GC.

**Electronic supplementary material:**

The online version of this article (10.1186/s12893-019-0593-6) contains supplementary material, which is available to authorized users.

## Background

The controlling nutritional status (CONUT) score was developed several years as an accessible nutritional screening tool for evaluating patients’ nutritional status calculated from serum albumin level, total cholesterol level, and total lymphocyte count [1]. As the prognostic role of nutritional status has been reported in the disease progression and survival of cancer patients [2], interest in the CONUT score increased. This led to recent reviews that showed the clinical evidence of the CONUT score on long-term prognosis in patients with gastrointestinal and hepato-pancreato-biliary cancer [3, 4]. In addition to these long-term associations, our research group has recently reported the association between the CONUT score and postoperative complication risk in gastrointestinal and hepato-pancreato-biliary surgical oncology [5, 6]. To date, however, published reviews did not restrict search strategy to organ-specific cancers or procedures. Different cancers and procedures are associated with different surgical complication risk and cancer prognosis. Therefore, the impact of the CONUT score in specific in cancers forming in specific organs, treated with specific surgical techniques, should be examined.

The impact of the CONUT score on outcome in patients with gastric cancer (GC) was first reported in 2018 [7]. After that, several studies on the CONUT score in GC have been published [8, 9]. However, results of these studies are reported differently, using different cut-off values of the CONUT score. To date, the impact of the CONUT score on outcomes has not yet been systematically examined in patients with GC.

The aim of the present study was to investigate the association between the CONUT score and outcomes including short-term and long-term prognosis in patients following gastrectomy for GC. Moreover, we explored the significance of the CONUT score on postoperative clinico-pathological parameters in patients with GC.

## Methods

The present study was reported according to the Preferred Reporting Items for Systematic Reviewers and Meta-Analyses (PRISMA) guidelines [10]. Details of the systematic literature search strategy have been reported previously [5]. A systematic literature search was conducted on the 15th of January 2019 to identify all available manuscripts that report the association between the CONUT score and outcomes in patients with GC (Additional file 1: Table S1). Inclusion criteria were the following: (1) patients undergoing curative resection for GC; (2) in whom the CONUT score was assessed preoperatively; and for whom (3) postoperative outcomes including short-term outcomes and long-term survival were reported.

After removing duplicate records, abstracts and full-text articles were screened independently by two investigators (KT and PD). The extracted data included year and country of study publication, study type, patient information, pathological finding, cut-off value of the CONUT score, and short-term and long-term outcomes. The Newcastle-Ottawa quality assessment scale for cohort studies was used to assess the methodological quality of each studies [11]. As previously, studies with a total score of 6 or higher were considered high-quality studies [12].

The primary endpoint of this report was long-term survival, and included overall survival (OS), cancer-specific survival (CSS) and recurrence free survival (RFS). Secondary endpoints were short-term outcomes: postoperative morbidity, mortality and postoperative clinico-pathological parameters. Postoperative complications and pathological findings were evaluated based on the definition proposed in each study. The postoperative clinico-pathological parameters included tumor (T) stage (T1/2 versus T3/4), lymph node (N) stage (N0/1 versus N2/3), TNM stage (stage I/II versus III), tumor differentiation (well differentiated versus poorly differentiated), and microvascular invasion (absent versus present).

### Statistical analysis

Analyses were performed using R 3.5.4 (cran.r-project.org) and Review Manager 5.3 (Cochrane Collaboration, 2014). The pooled hazard ratios (HR) and odds ratios (OR) with 95% confidence interval (95%CI) were calculated for the different outcomes using the inverse variance method. Heterogeneity among studies was quantified by calculating the *I*^*2*^ values and a *Χ*^2^ test was conducted, with *P* < 0.05 being statistically significant and *I*^*2*^ values of 50% or more indicating the presence of heterogeneity. We conducted random effects meta-analyses for all outcomes, as studies were heterogeneous with regards to population and received treatments. Funnel plots were utilized in order to evaluate potential publication bias for all reviewed outcomes.

## Results

**Fig. 1:**
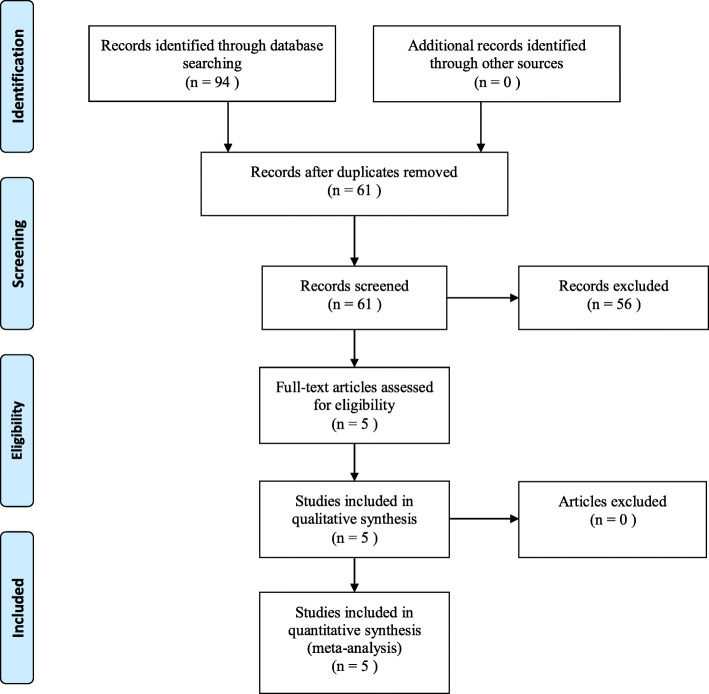
PRISMA 2009 Flow Diagram

The literature search according to the PRISMA guidelines identified five studies as shown in Fig. 1 [7–9, 13, 14]. Five articles with 2482 patients were included. All studies were retrospective series from Asian countries, more particularly Japan and China in 2018 (Table 1). Four [7–9, 14] were single center studies and one [13] was multi-center study. Different cut-off values of the CONUT score were used in each study. All studies focused on long-term survival such as OS, CSS, or RFS. Median follow-up period ranged between 36 and 61 months. The quality assessment of the included studies showed that all studies were considered to be high-quality (Additional file 1: Table 2). Funnel plots of OS, CSS, and RFS demonstrated no obvious asymmetry (Additional file 1: Figure S1). Regarding surgical procedures, 723 out of 2066 (35.0%) were total gastrectomy, and 1343 (65.0%) were partial or subtotal gastrectomy [8, 9, 13, 14]. In contrast, 73.6% (1520 of 2066) were open procedure and 26.4% (546 of 2066) were laparoscopic procedure.

**Table 1:** Literatures investigation the effects of the CONUT score in patients undergoing curative resection for gastric cancer

Study	Year	Country	Study design	Number (Male)	Tumor stage	Cut-off for high CONUT group	Prevalence of high CONUT score	End points	Follow-up (median, months)	Quality^a^
Kuroda [7]	2018	Japan	Retrospective Single center	416 (267)	I: 275 II: 81 III: 60	≥4	14.9%	OS CSS RFS Complications	61.2	6
Zheng [8]	2018	China	Retrospective Single center	532 (403)	I: 165 II: 123 III: 244	0–1 (normal) 2–4 (light) ≥5 (moderate or severe)	54.7% 34.4% 10.9%	OS RFS	60	7
Liu [9]	2018	China	Retrospective Single center	697 (457)	II: 194 III: 503	≥3	31.1%	CSS Complications	36	6
Ryo [13]	2018	Japan	Retrospective Multi-center	626 (435)	II: 281 III: 345	≥2	46.2%	OS RFS Complications	49.2	7
Suzuki [14]	2018	Japan	Retrospective Single center	211 (141)	I: 132 II: 53 III: 26	≥5	17.1%	OS CSS Complications	47	6

^a^Score from a maximum of 9 evaluated by the Newcastle–Ottawa quality assessment scale for cohort studies. [11]

*CONUT* Controlling nutritional status, *OS* Overall survival, *CSS* Cancer-specific survival, *RFS* Recurrence/relapse-free survival

**Table 2:** Studies reporting the effects of the CONUT score on outcomes in patients with gastric cancer

Study	Overall complication	Mortality	Reccurence/relapse-free survival	Cancer-specific survival	Overall survival
Kuroda [7]	37.1 vs 27.7% (*P* = 0.133)	n.a.	5-year: 77.8 vs 90.6% (*P* = 0.017) HR 2.63 (1.16–5.98), *P* = 0.021**	5-year: 82.3 vs 94.0% (*P* = 0.019) HR 4.13 (1.62–10.55), *P* = 0.003**	5-year: 43.8 vs 84.8% (*P* < 0.001) HR 2.72 (1.74–4.25), *P* < 0.001*
Zheng [8]	n.a.	n.a.	5-year: 51.6 vs 70% (Light vs normal) (*P* < 0.001) HR 1.376 (1.005–1.884)* 55.2 vs 70% (Moderate vs normal) (*P* = 0.017) HR 1.154 (0.726–1.836), *P* = 0.137*	n.a.	5-year: 53.2 vs 71.4% (Light vs normal) (*P* < 0.001) HR 1.360 (0.984–1.879)* 54.5 vs 71.4% (Moderate vs normal) (*P* = 0.006) HR 1.266 (0.753–2.126), *P* = 0.173*
Liu [9]	26.7 vs 21.9% (*P* = 0.161)	n.a.	n.a.	5-year: 39.3 vs 55.5% (*P* < 0.001) HR 1.553 (1.080–2.232), *P* = 0.017*	n.a.
Ryo [13]	31.5 vs 26.4%	90-day: 1.4 vs 0.3%	HR 1.33 (0.98–1.81), *P* = 0.0637**	n.a.	HR 1.74 (1.26–2.41), *P* = 0.0007*
Suzuki [14]	55.5 vs 36% (*P* = 0.09) Infectious: 44.4 vs 25.7% (*P* = 0.08) OR 2.36 (0.99–5.40), *P* = 0.046*	3 vs 0% (*P* = 0.09)	n.a.	5-year: 64, 75 vs 33% (stage II/III; normal, light vs moderate and severe) (*P* = 0.003) HR 3.75 (1.30–10.43), *P* = 0.015*	5-year: 88, 76 vs 51% (stage I; normal, light vs moderate and severe) (*P* = 0.044) 64, 53 vs 24% (stage II/III; normal, light vs moderate and severe) (*P* = 0.007) HR 2.12 (1.18–3.69), *P* = 0.012*

Data are shown for high CONUT group versus low CONUT group unless otherwise indicated. OR and HR is shown with 95% confidence interval. *Multivariable analysis. **Univariate analysis

*CONUT* Controlling nutritional status, *HR* Hazard ratio, *OR* Odds ratio, *n.a*., not available

**Fig. 2:**
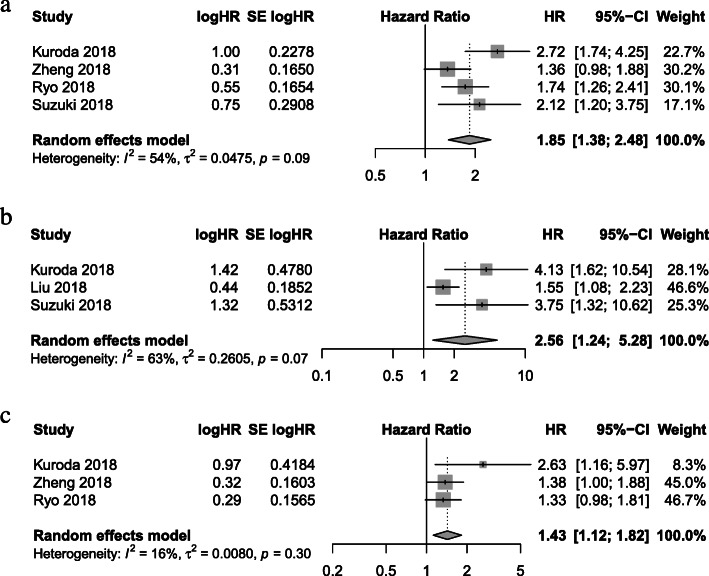
Forest plots demonstrating primary endpoint in terms of high CONUT group versus low CONUT group. **a** Overall survival; **b** Cancer-specific survival; and **c** Recurrence/relapse-free survival

Table 2 details the correlation between the CONUT score and the different outcomes of interest in the different studies. The results of meta-analysis for the primary and secondary endpoints in terms of high CONUT group versus low CONUT group are shown in Fig. 2 and Table 3 respectively.

**Table 3:** Secondary endpoints in terms of high CONUT group versus low CONUT group

Variables	No. of studies	No. of patients	OR	95%CI	*P* value	*I*^*2*^ (%)	Heterogeneity P value
Overall complications	4	1950	1.39	1.12–1.74	0.003	0	0.56
Mortality	2	837	6.79	1.10–41.7	0.04	0	0.56
T3/4	3	1739	1.75	1.30–2.34	< 0.001	0	0.47
N2/3	3	1739	1.51	1.20–1.90	< 0.001	5	0.35
Stage III	5	2482	1.73	1.29–2.33	< 0.001	55	0.07
Poor differentiation	5	2482	0.85	0.68–1.05	0.13	16	0.31
Microvascular invasion	3	1645	1.50	1.02–2.00	0.006	0	0.64

*CONUT* Controlling nutritional status, *OR* Odds ratio, 95%CI, 95% confidence interval

The impact of the CONUT score on OS was investigated in four studies. All studies reported 5-year OS in the high CONUT group was significantly poorer than that in the low CONUT group [7, 8, 13, 14]. The multivariable analysis of each study revealed that the CONUT score was as an independent predictor associated with OS. Our meta-analysis within these studies comprising 1785 patients demonstrated that the CONUT score was associated with OS (HR 1.85, 95%CI 1.38–2.48, *P* < 0.001, *I*^*2*^ = 54%, *P* = 0.09) (Fig. 2a).

The relationship between the CONUT score and CSS was reported in three studies [7, 9, 14]. In two studies the CONUT score was an independent prognostic factor for CSS in the multivariable analysis [9, 14], however one study showed no significant association between the CONUT score and CSS [7]. Meta-analysis of outcomes within these studies identified an association between the CONUT score and CSS (HR 2.56, 95%CI 1.24–5.28, *P* = 0.01, *I*^*2*^ = 63%, *P* = 0.07, *n* = 1324) (Fig. 2b).

Three studies [7, 8, 13] reported the influence of the CONUT score on RFS. The multivariable analysis of each study showed no significant association between the CONUT score and RFS. However, our meta-analysis indicated that the CONUT score was associated with RFS (HR 1.43, 95%CI 1.12–1.82, *P* = 0.004, *I*^*2*^ = 16%, *P* = 0.30, *n* = 1574) (Fig. 2c).

The effect of the CONUT score on postoperative overall complications was reported in four studies, demonstrating no significant association between the CONUT score and postoperative complications [7, 9, 13, 14]. One study conducted the multivariable analysis showing that high CONUT score was an independent risk factor for procedure-unrelated infectious morbidity (OR 2.36, 95%CI 0.99–5.40, *P* = 0.046) [14]. The two studies reported the no correlation between the CONUT score and postoperative mortality [13, 14]. In meta-analysis, the CONUT score was found to be associated with the incidence of postoperative overall complications (OR 1.39, 95%CI 1.12–1.74, *P* = 0.003, *I*^*2*^ = 0%, *P* = 0.56, *n* = 1950) and mortality (OR 6.97, 95%CI 1.10–41.7, *P* = 0.04, *I*^*2*^ = 0%, *P* = 0.56, *n* = 837).

Regarding the postoperative clinico-pathological parameters, the CONUT score was associated with T stage (OR 1.75, 95%CI 1.30–2.34, *P* < 0.001, *I*^*2*^ = 0%, *P* = 0.47, *n* = 1739), N stage (OR 1.51, 95%CI 1.20–1.90, *P* < 0.001, *I*^*2*^ = 5%, *P* = 0.35, *n* = 1739), TNM stage (OR 1.73, 95%CI 1.29–2.33, *P* < 0.001, *I*^*2*^ = 55%, *P* = 0.07, *n* = 2482), and microvascular invasion (OR 1.50, 95%CI 1.02–2.00, *P* = 0.006, *I*^*2*^ = 0%, *P* = 0.64, *n* = 1645). However, no significant association was found in tumor differentiation (OR 0.85, 95%CI 0.68–1.05, *P* = 0.13, *I*^*2*^ = 16%, *P* = 0.31, *n* = 2482).

## Discussion

The present study suggests that the CONUT score is a potential nutritional screening tool for the prediction of the outcomes including not only long-term and short-term outcomes, but also clinico-pathological parameters in patients undergoing gastrectomy for GC. To the best of our knowledge, this systematic review and meta-analysis is the first to investigate the prognostic impact of the CONUT score on outcomes in patients with GC. The present study demonstrated that the CONUT score was associated with long-term prognosis and the incidence of postoperative complications and mortality after gastrectomy for GC. Furthermore, we found the significant association between the CONUT score and clinico-pathological parameters.

A recent study has shown the CONUT score could be a prognostic indicator of long-term survival in gastrointestinal cancer patients after surgery, however this analysis included only one article in patients with GC [4]. Therefore, the authors suggested to explore the prognostic value of the CONUT score specifically for the different included cancers. In contrast, the prognostic nutritional index (PNI), calculated based on the serum albumin level and total lymphocyte count, has well described correlations with short-term and long-term outcomes in the literature [15]. In a recent meta-analysis, the prognostic significance of the PNI for prognosis in patients with cancers was confirmed [16]. Furthermore, the significance of PNI in patients with GC has been investigated in a review with ten studies describing 3396 patients [17]. Several studies have reported that the CONUT score was a better prognostic index of short-term and long-term outcome than the PNI in various cancers [6, 9, 13, 18–20]. Furthermore, the CONUT score has been reported to be the most accurate predictor compared with other prognostic factors such as the neutrophil to lymphocyte ratio, modified Glasgow Prognostic Score, tumor stage, and immune-nutritional factors [7, 20]. Our findings confirm clinical significance of the CONUT score in patients with GC.

In the present study, we analyzed the results of five published retrospective studies on subjects with the CONUT score and outcomes of GC (Table 2). Although some studies showed the association between the CONUT score and OS, the relationship between the CONUT and CSS, RFS, and the risk of postoperative complication remained controversial because of differences in reported outcomes (Table 3). Moreover, the significance of the CONUT score on clinico-pathological parameters was unknown. The present meta-analysis indicated that patients with high CONUT score had a significantly worse OS, CSS and RFS, and had a higher incidence of postoperative complications and mortality in patients with GC. Furthermore, high CONUT score was significantly associated with more advanced tumor characteristics including advanced T and N stage, advanced TNM stages, and positive microvascular invasion.

The biological mechanism regarding the association between the CONUT score and outcomes has not been fully investigated in GC patients, however there were several reasons to explain why a high CONUT score was associated with poor outcomes in GC. First, each of components of the CONUT score has been reported to be related to outcomes in patients with GC. Serum albumin is a major indicator of nutritional status and systematic inflammation and is reported to be associated with the survival in patients with GC [21, 22]. Serum cholesterol level has been reported to correlate with tumor progression and survival in various gastrointestinal cancers [23–25]. Total lymphocyte, an indicator of immune and nutritional status in cellular and antiviral immunity, is reported to be related with the prognosis in GC [26]. Secondly, our results suggested that high CONUT score was associated with more advanced tumor characteristics, including advanced T and N stage, advanced TNM stages, and positive microvascular invasion. Although the association between the PNI and the extent of tumor progression has been reported [17], it remains unknown whether the results of the CONUT score or PNI were a cause or a consequence of tumor progression. Finally, emerging evidence has shown that patients’ frailty evaluated by sarcopenia was associated with the prognosis and the risk of postoperative complications in gastrointestinal surgical oncology [27, 28]. Zheng et al. reported that sarcopenia was a more objective predictor than the CONUT score of the survival in GC, although this finding must be confirmed by a large prospective study [8].

We acknowledge several limitations of the present study. The number of included studies in the meta-analysis was relatively small. All the included studies were retrospective cohort study from Asian countries, therefore it remains unknown whether our results can be applied to Western populations. Future studies should be validated in Western population. Different cut-off values were used in each studies. Although the original CONUT score article described four categories, the cut-off values of the CONUT score used in the literature differs between and different cancers [3, 4]. Finally, the nature of the relationship between the CONUT score and outcomes is not fully elucidated in patients with GC. Therefore, further well-designed studies are needed to identify the impact of the CONUT score on outcomes and to determine the most appropriate cut-off value to predict the survival and complication risks in patients with GC.

In conclusion, the present study suggests that the preoperative CONUT score could be an indicator to predict the survival, postoperative complications and postoperative clinico-pathological parameters in patients following gastrectomy for GC. This review highlights the need for comprehensive assessment of the CONUT score in clinical practice as it would be helpful for decision-making in patients with GC. Evaluating the CONUT score is easy and practical to estimate the prognosis and complication risks, therefore the CONUT score should be evaluated preoperatively in patients undergoing gastrectomy for GC.

## Additional file

Additional file 1: **Table S1.** Search strings and terms. **Table S2.** The Newcastle-Ottawa scale for quality assessment of include studies. **Figure S1.** Funnel plots demonstrating primary endpoint in terms of low CONUT versus high CONUT score. (a) OS; (b) CCS; and (c) RFS. (DOCX 110 kb)

## Data Availability

The data supporting the conclusions of this article are included in this published article.

## Abbreviations

95%CI: 95% confidence interval
CONUT: Controlling nutritional status
CSS: Cancer-specific survival
GC: Gastric cancer
HR: Hazard ratio
OR: Odds ratio
OS: Overall survival
RFS: Recurrence/relapse-free survival
