# Supplementary material for: Prognostic significance of the controlling nutritional status (CONUT) score in patients undergoing gastrectomy for gastric cancer: a systematic review and meta-analysis
Source: BMC Surg. 2019 Sep 5;19:129. doi: 10.1186/s12893-019-0593-6 (PMC6729085; doi:10.1186/s12893-019-0593-6)

**Additional file 1**

**Supplementary Table 1.** Search strings and terms

| **Database** | **Search query** | **Number of found records** | **Number of found records without duplication** |
| --- | --- | --- | --- |
| Embase.com | ('controlling nutritional status score'/de OR 'controlling nutritional status'/de OR ('serum albumin'/de AND 'cholesterol blood level'/exp AND 'lymphocyte count'/exp) OR ('Controlling Nutritional Status' OR conut OR (albumin NEAR/10 cholesterol NEAR/10 lymphocyte NEAR/10 (count* OR serum* OR concentrat*))):ab,ti) AND ('stomach tumor'/exp OR 'gastrectomy'/exp OR (((gastric* OR stomach) NEAR/3 (tumor* OR tumour* OR cancer* OR malign* OR carcinom* OR adenocarcinom* OR surg* OR resect*)) OR gastrectom* ):ab,ti) | 27 | 26 |
| Medline Ovid | ((Serum Albumin/ AND exp cholesterol/bl AND exp Lymphocyte Count/) OR (Controlling Nutritional Status OR conut OR (albumin ADJ10 cholesterol ADJ10 lymphocyte ADJ10 (count* OR serum* OR concentrat*))).ab,ti.) AND (Stomach Neoplasms/ OR gastrectomy/ OR (((gastric* OR stomach) ADJ3 (tumor* OR tumour* OR cancer* OR malign* OR carcinom* OR adenocarcinom* OR surg* OR resect*)) OR gastrectom* ).ab,ti.) | 18 | 2 |
| Web of science | TS=((("Controlling Nutritional Status" OR conut OR (albumin NEAR/10 cholesterol NEAR/10 lymphocyte NEAR/10 (count* OR serum* OR concentrat*)))) AND ((((gastric* OR stomach) NEAR/2 (tumor* OR tumour* OR cancer* OR malign* OR carcinom* OR adenocarcinom* OR surg* OR resect*)) OR gastrectom* ))) | 16 | 8 |
| Cochrane CENTRAL | (('Controlling Nutritional Status' OR conut OR (albumin NEAR/10 cholesterol NEAR/10 lymphocyte NEAR/10 (count* OR serum* OR concentrat*))):ab,ti) AND ((((gastric* OR stomach) NEAR/3 (tumor* OR tumour* OR cancer* OR malign* OR carcinom* OR adenocarcinom* OR surg* OR resect*)) OR gastrectom* ):ab,ti) | 3 | 1 |
| Google scholar | "Controlling Nutritional Status"\|conut "gastric\|stomach tumor\|tumour\|cancer\|malignancy\|carcinoma\|resection"\|gastrectomy | 30 | 24 |
| Total |  | **94** | **61** |

**Supplementary Table 2.** The Newcastle-Ottawa scale for quality assessment of include studies.

| **Study** | **Selection** | | | | **Comparability** | **Outcome** | | | **Total score** |
| --- | --- | --- | --- | --- | --- | --- | --- | --- | --- |
|  | Representativeness  of the exposed  cohort | Selection  of the  non-exposed  cohort | Ascertainment  of exposure | Demonstration  that outcome  of interest was  not present at  start of study | Comparability  of cohorts on  the basis of  the design or  analysis | Assessment  of outcome | Was follow-  up long  enough for  outcomes to occur | Adequacy  of follow  up of  cohorts |  |
| Total score | 1 | 1 | 1 | 1 | 2 | 1 | 1 | 1 | 9 |
| Kuroda [7] | 1 | 1 | 1 | 0 | 0 | 1 | 1 | 1 | 6 |
| Zheng [8] | 1 | 1 | 1 | 0 | 1 | 1 | 1 | 1 | 7 |
| Liu [9] | 1 | 1 | 1 | 0 | 0 | 1 | 1 | 1 | 6 |
| Ryo [13] | 1 | 1 | 1 | 0 | 1 | 1 | 1 | 1 | 7 |
| Suzuki [14] | 1 | 1 | 1 | 0 | 0 | 1 | 1 | 1 | 6 |

**Supplementary Figure 1.** Funnel plots demonstrating primary endpoint in terms of low CONUT versus high CONUT score. (a) OS; (b) CCS; and (c) RFS.

a. OS


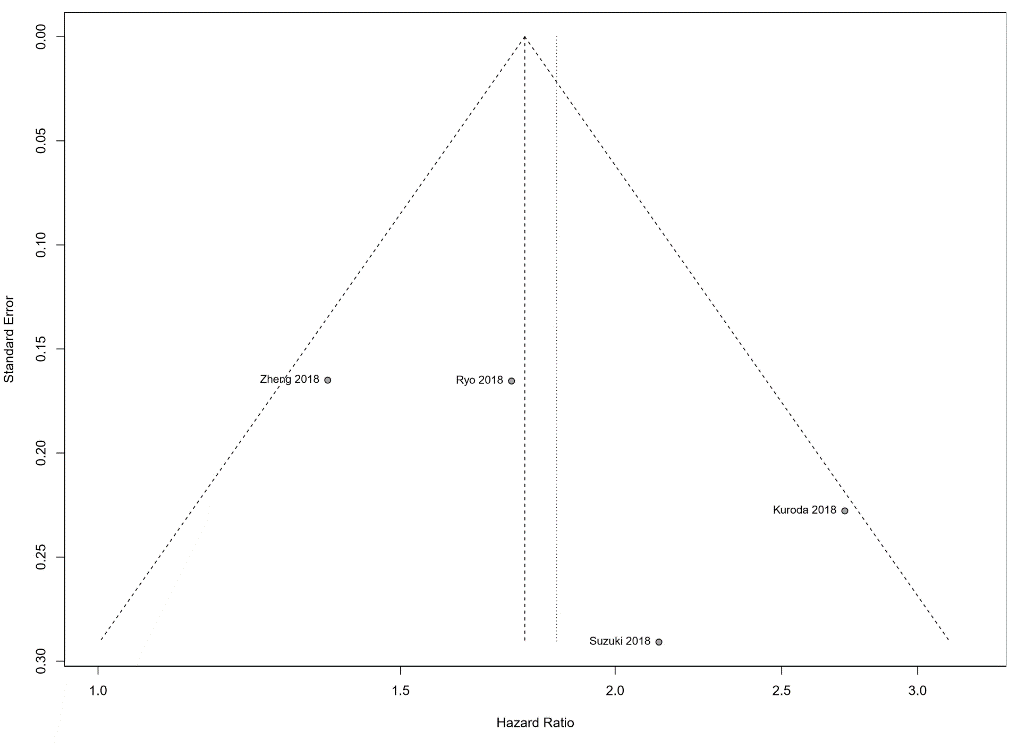


b. CCS


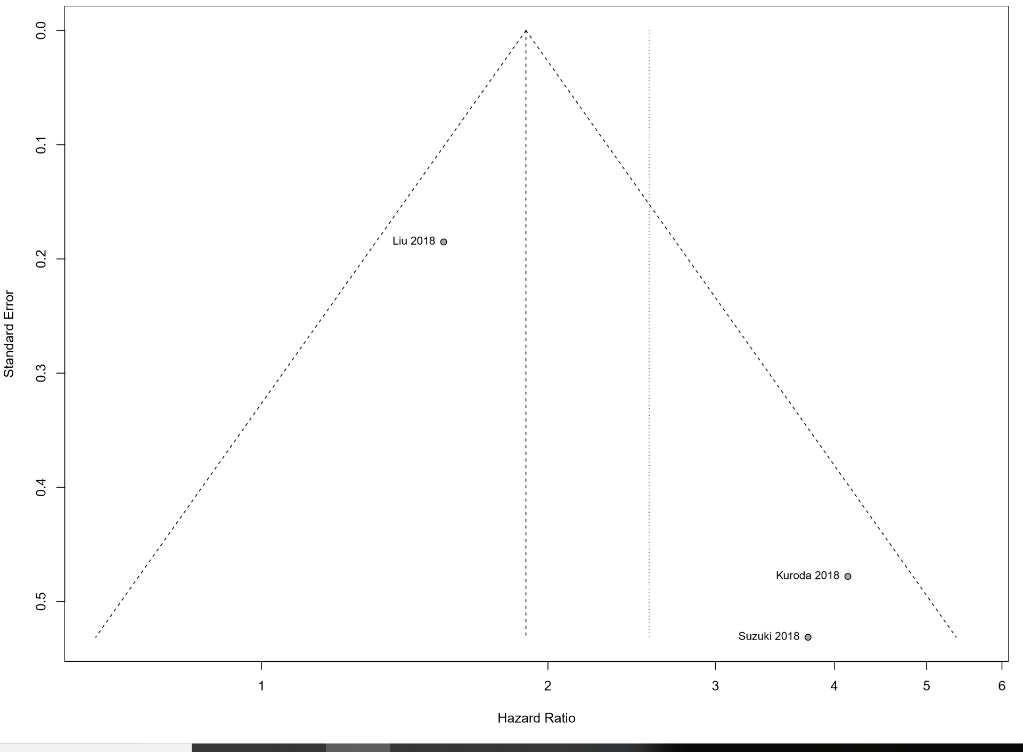


c. RFS


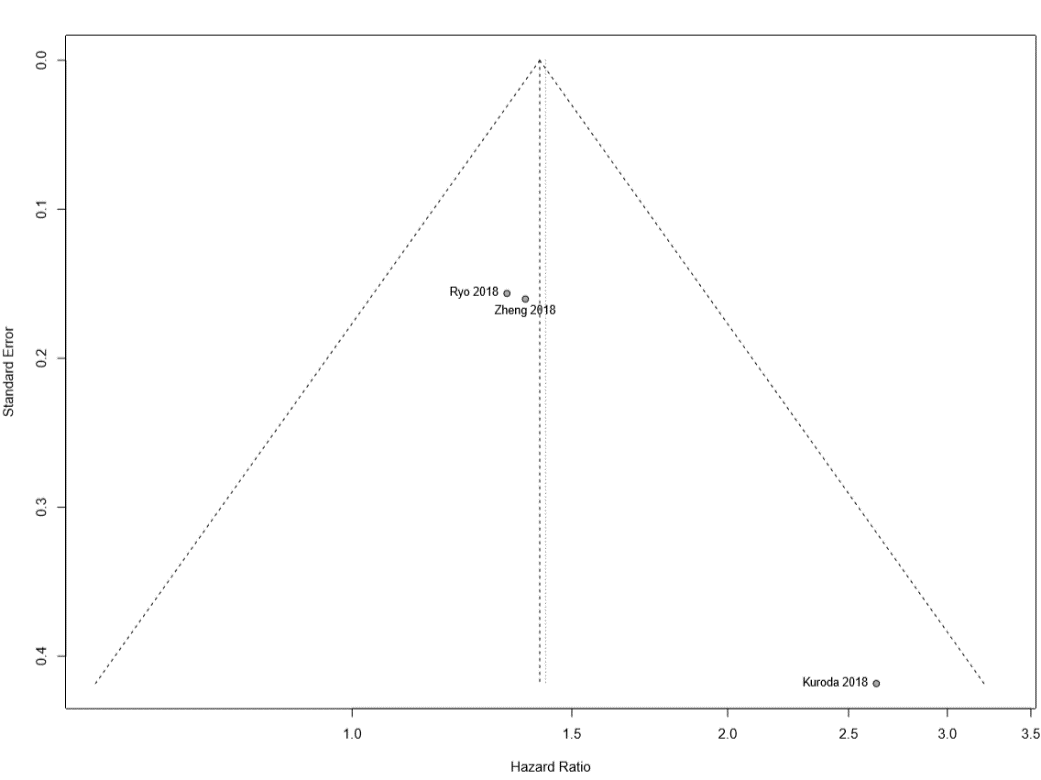

Supplement: Supplementary file 1 — Table S1. Search strings and terms. Table S2. The Newcastle-Ottawa scale for quality assessment of include studies. Figure S1. Funnel plots demonstrating primary endpoint in terms of low CONUT versus high CONUT score. (a) OS; (b) CCS; and (c) RFS. (DOCX 110 kb) [file 12893_2019_593_MOESM1_ESM.docx]
